# Supplementary material for: Response Surface Methodology to Efficiently Optimize Intracellular Delivery by Photoporation
Source: Int J Mol Sci. 2023 Feb 5;24(4):3147. doi: 10.3390/ijms24043147 (PMC9962540; doi:10.3390/ijms24043147)
Supplement: Supplementary file 1 [file ijms-24-03147-s001.zip › ijms-2149938-supplementary.pdf]

Supporting information

Response surface methodology to efficiently optimize intracellular delivery by photoporation

Ilia Goemaere [a,b], Deep Punj [a], Aranit Harizaj [a], Jessica Woolston [a], Sofie Thys [b], Karen Sterck [b], Stefaan C. De Smedt [a], Winnok H. De Vos [b, #] and Kevin Braeckmans [a,#,*]

[a] Laboratory of General Biochemistry and Physical Pharmacy, Faculty of Pharmaceutical Sciences, Ghent University, Ottergemsesteenweg 460, 9000 Ghent, Belgium

[b] Laboratory of Cell Biology and Histology, Department of Veterinary Sciences, University of Antwerp, Universiteitsplein 1, 2610 Antwerp, Belgium

[#]Shared senior authorship

*Correspondence: Kevin.Braeckmans@ugent.be; Tel.: +32-9-2648098; Fax: +32-9-2648189

Table S1. Summary of the average hydrodynamic diameter (size), polydispersity index (PDI) and zeta potential (Z.P.) of the BSA-coated PDNPs with sizes corresponding to factor levels -1.789, -0.5, 0.25 and 1.789 in both HyClone Pure water and Opti-MEM. Z.P. was only measured for BSA-coated PDNPs in HyClone Pure water.

	**142 nm**<br>**Factor level: -1.789**		**400 nm**<br>**Factor level: -0.5**		**550 nm**<br>**Factor level: 0.25**		**858 nm**<br>**Factor level: 1.789**	
	HyClone	Opti-MEM	HyClone	Opti-MEM	HyClone	Opti-MEM	HyClone	Opti-MEM
Size (nm)	135.3 ± 1.9	135.6 ± 0.4	394.4 ± 1.6	400.2 ± 3.9	546.6 ± 16.2	588.1 ± 6,8	821.2 ± 16.5	797.4 ± 9.0
PDI	0.062 ± 0.003	0.045 ± 0,004	0.018 ± 0.016	0.018 ± 0,024	0.091 ± 0.042	0.038 ± 0.043	0.112 ± 0.026	0.033 ± 0.012
Z.P. (mV)	- 29.5 ± 0.5		- 26.8 ± 0.5		- 11.4 ± 0.5		-22.3 ± 0.3	

Figure S1. Representative intensity size distributions as measured by DLS of BSA coated PDNPs of 142 nm (A), 400 nm (B), 550 nm (C) and 858 nm (D) in water (blue) and 15 min incubated in Opti-MEM (red).

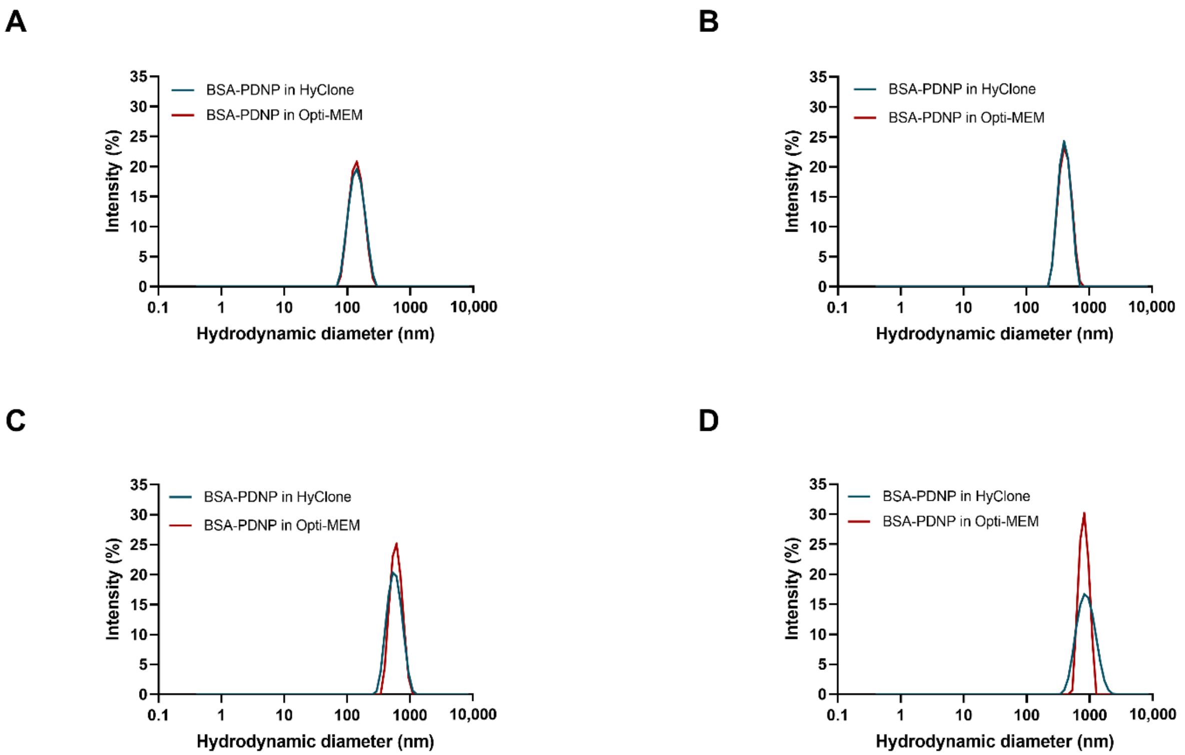

Table S2. Summary of the average (±standard deviation) hydrodynamic diameter (size) and polydispersity index (PDI) of the BSA coated PDNPs for all sizes used in this study on both day 1 of their synthesis and day 90 to illustrate the long-term colloidal stability of the PDNPs.

	**Day 1**		**Day 90**	
**BSA-coated PDNP nominal size**	**Size (nm)**	**PDI**	**Size (nm)**	**PDI**
142 nm	135.3 ± 1.9	0.062 ± 0.003	135.2 ± 2.9	0.081 ± 0.020
300 nm	290.8 ± 3.5	0.084 ± 0.020	293.0 ± 5.9	0.056 ± 0.019
400 nm	394.4 ± 1.6	0.018 ± 0.016	396.6 ± 7.2	0.050 ± 0.023
500 nm	516.1 ± 9.3	0.051 ± 0.014	503.7 ± 14.0	0.028 ± 0.022
550 nm	546.6 ± 16.2	0.091 ± 0.042	545.5 ± 7.4	0.068 ± 0.054
700 nm	728.4 ± 8.7	0.118 ± 0.048	742.1 ± 24.8	0.102 ± 0.050
858 nm	821.2 ± 16.5	0.112 ± 0.026	837.4 ± 16.0	0.148 ± 0.044

Table S3. Summary of the average (±standard deviation) nanoparticle diameter of the BSA coated PDNPs for all sizes used in this study as measure by both scanning electron microscopy (SEM) and transmission electron microscopy (TEM).

**BSA-coated PDNP nominal size**	**SEM**<br>**Nanoparticle diameter (nm)**	**TEM**<br>**Nanoparticle diameter (nm)**
142 nm	94 ± 11	96 ± 13
300 nm	202 ± 23	208 ± 32
400 nm	321 ± 29	332 ± 50
500 nm	359 ± 48	364 ± 44
550 nm	366 ± 40	366 ± 30
700 nm	471 ± 70	467 ± 84
858 nm	515 ± 56	570 ± 80

Figure S2. PDNP size distributions as determined by electron microscopy for the relevant PDNP sizes represented in Figure 3 in main manuscript. (A-C) Size distributions for (A) 300 nm, (B) 500 nm and (C) 700 nm PDNPs as derived from SEM images. (D-F) Size distributions for (D) 300 nm, (E) 500 nm and (F) 700 nm PDNPs as derived from TEM images.

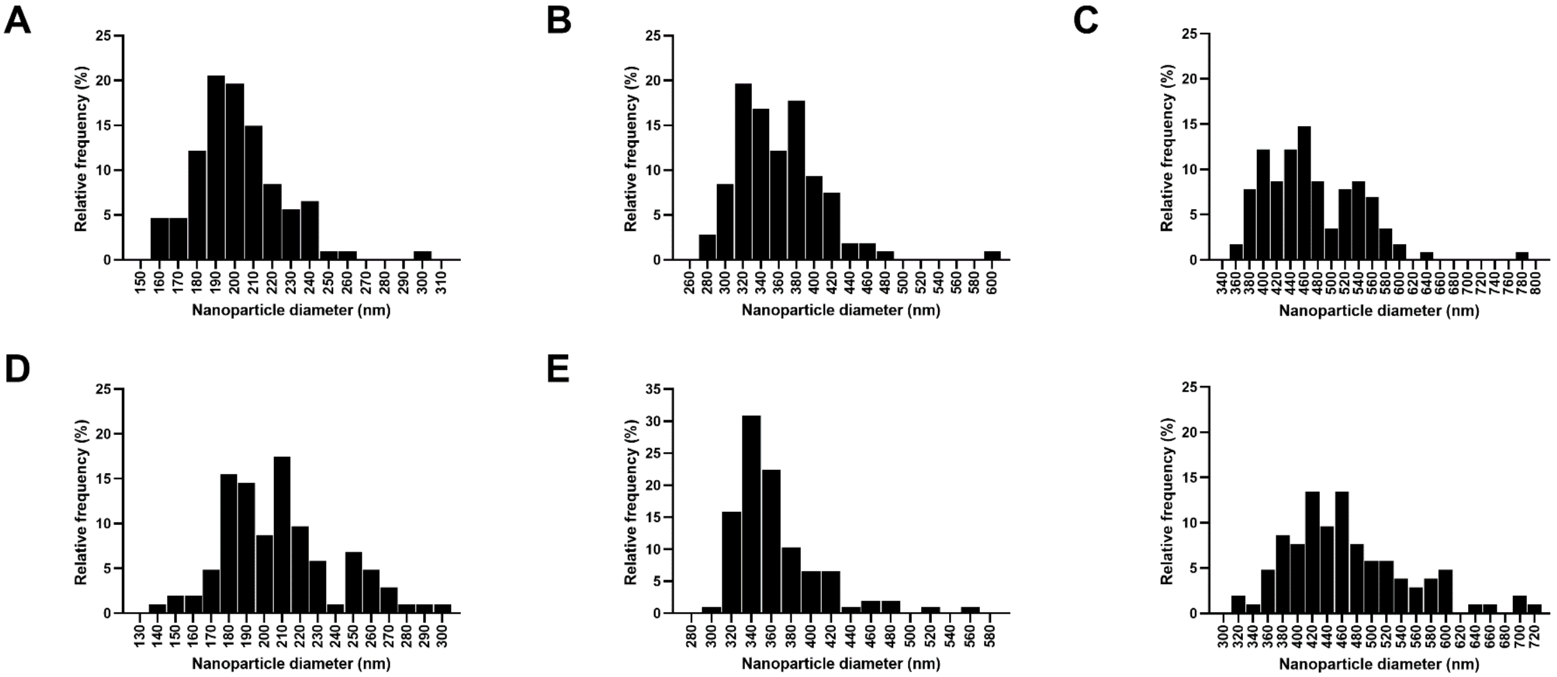

Table S4. Model estimates and Analysis of variance (ANOVA) for the optimization of photoporation yield using PDNPs as photothermal NPs and FD500 as cargo molecule. with both a CCC design and BBD. Triplicates were performed to assess model output reproducibility. Values indicated in red and with * are significant at $p < 0.05$.

	**CCC**						**BBD**					
	**Replicate 1**		**Replicate 2**		**Replicate 3**		**Replicate 1**		**Replicate 2**		**Replicate 3**	
**Term**	**Estimate**	**p-value**	**Estimate**	**p-value**	**Estimate**	**p-value**	**Estimate**	**p-value**	**Estimate**	**p-value**	**Estimate**	**p-value**
Intercept	41.452	$0.916e^{-8}$ *	38.532	$0.413e^{-7}$ *	35.526	$0.298e^{-8}$ *	36.939	$5.874e^{-7}$ *	42.567	$2.684e^{-7}$ *	40.077	$8.981e^{-7}$ *
Size	3.589	0.059	4.411	0.044 *	3.714	0.015 *	5.028	0.006 *	3.753	0.021 *	3.432	0.048 *
Conc.	1.007	0.555	0.131	0.945	0.346	0.782	2.530	0.078	-1.327	0.312	-0.828	0.573
Fluence	-1.027	0.549	-1.916	0.330	-1.440	0.268	1.030	0.420	-0.157	0.901	-1.845	0.232
Size*Conc.	-2.999	0.208	-1.524	0.556	-3.004	0.101	-1.690	0.354	-0.750	0.675	-5.746	0.026 *
Size*Fluence	-1.539	0.502	-1.253	0.627	0.817	0.628	-1.688	0.355	-4.013	0.056	-1.695	0.421
Conc.*Fluence	-0.132	0.953	0.353	0.890	-1.181	0.488	-0.144	0.934	-1.729	0.349	-2.066	0.333
$Size^2$	-10.199	$2.000e^{-4}$ *	-10.236	$5.000e^{-4}$ *	-9.635	$0.379e^{-6}$ *	-16.784	$5.912e^{-5}$ *	-17.927	$4.296e^{-5}$ *	-17.196	$1.000e^{-4}$ *
$Conc.^2$	-2.686	0.130	-4.858	0.027 *	-4.097	0.008 *	-3.548	0.080	-0.032	0.985	-0.550	0.789
$Fluence^2$	-3.906	0.040 *	-2.121	0.273	-3.180	0.027 *	-1.537	0.397	-0.470	0.791	0.076	0.970
**Statistic**	**F-value**	**p-value**	**F-value**	**p-value**	**F-value**	**p-value**	**F-value**	**p-value**	**F-value**	**p-value**	**F-value**	**p-value**
Model	5.516	0.012 *	4.616	0.021 *	9.301	0.002 *	14.41	0.002 *	14.32	0.002 *	10.61	0.004 *
Lack-of-fit	1.362	0.425	4.586	0.120	3.334	0.175	2.550	0.231	5.5801	0.096	1.460	0.382

Table S5. Summary statistics for the optimization of photoporation yield using PDNPs as photothermal NPs and FD500 as cargo molecule. with both a CCC design and BBD. Triplicates were performed to assess model output reproducibility. Values indicated in red and with * are significant at $p < 0.05$.

	**CCC**			**BBD**		
	**Replicate 1**	**Replicate 2**	**Replicate 3**	**Replicate 1**	**Replicate 2**	**Replicate 3**
**Test**	**p-value**	**p-value**	**p-value**	**p-value**	**p-value**	**p-value**
Shapiro-Wilk	0.200	0.581	0.160	0.421	0.661	0.979
Bruesch-Pagan	0.1182	0.076	0.108	0.357	0.118	0.588
Durbin-Watson	0.208	0.906	0.480	0.914	0.214	0.062

Figure S3. Representative Q-Q plots and residual vs. run order plots. (A) Q-Q plot for model fitted to replicate 2 of the CCC design (B) Q-Q plot for model fitted to replicate 2 of the BBD (C) Residual vs. run order plot for model fitted to replicate 2 of the CCC design (D) Residual vs. run order plot for model fitted to replicate 2 of the BBD.

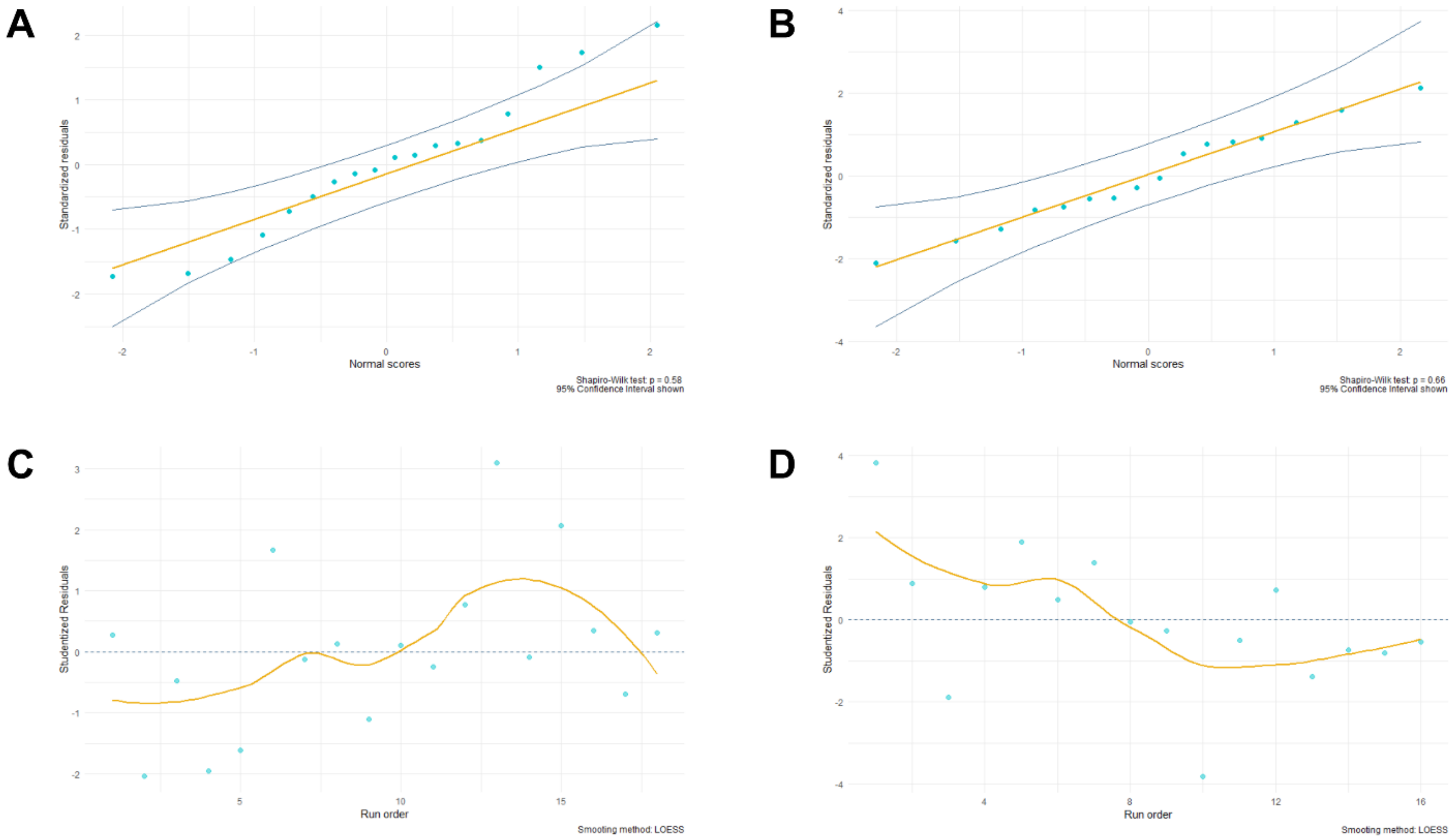

Figure S4. Representative response surfaces where one factor was kept constant and the other factors varied. Regardless of the (A) PDNP concentration. (C) PDNP size or (E) laser fluence that was kept constant. the CCC design-based models indicated the presence of an optimum. In case of the BBD-based models. a ridge system was present when the (B) PDNP concentration. (D) PDNP size or (F) laser fluence was kept constant while varying the other factors.

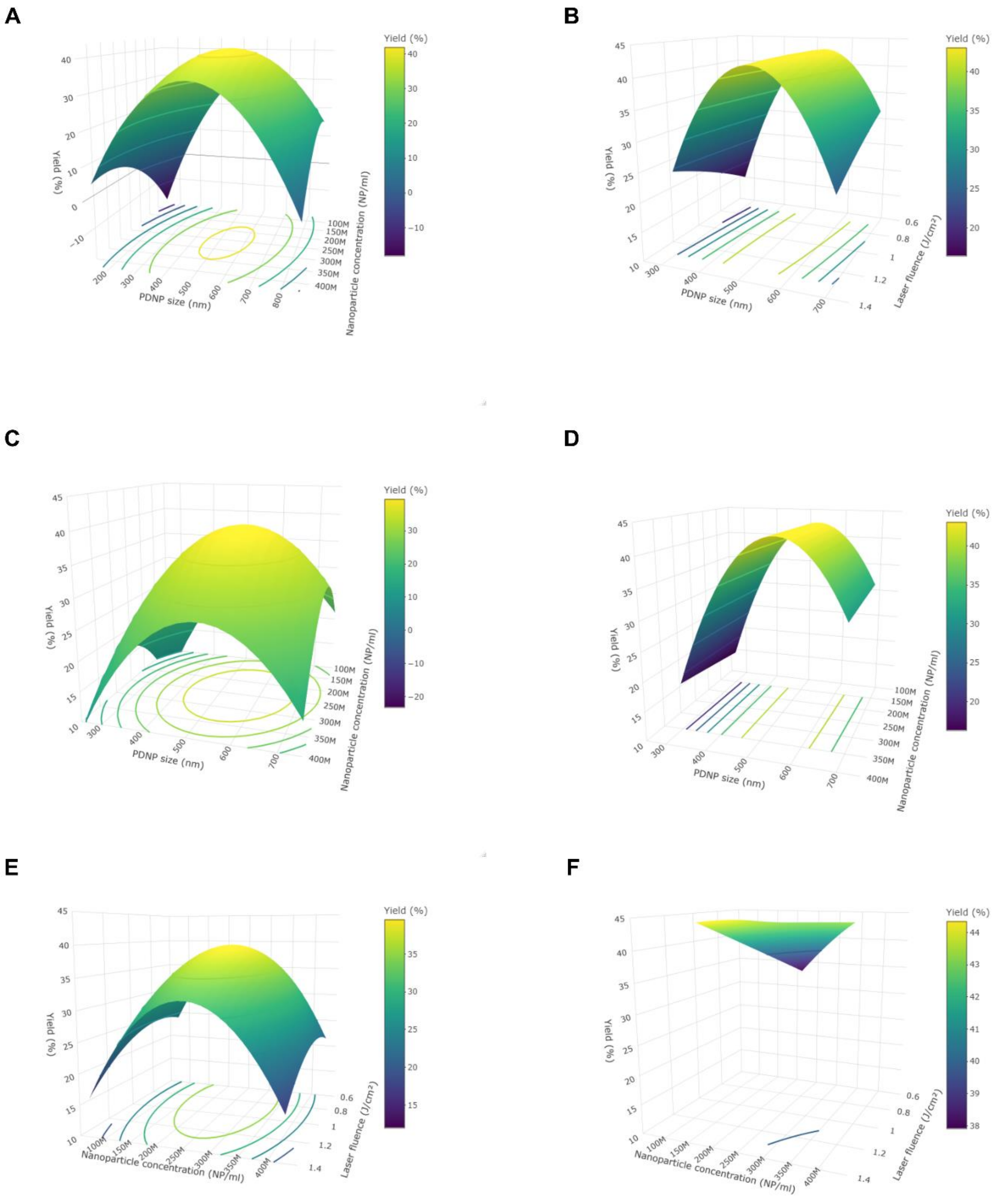

Table S6. Overview of the parameter values included in the BBD with extended variable ranges and their translation to coded values as they are used in the RSM model.

	**Parameter values**		
**Size (nm)**	300	500	700
**Concentration (NPs/ml)**	$1.0*10^8$	$3*10^8$	$5.0*10^8$
**Fluence (J/cm²)**	0.5	1	1.5
**Coded factor levels**	**-1**	**0**	**+1**

Table S7. Model estimates and Analysis of variance (ANOVA) for the optimization of photoporation yield using PDNPs as photothermal NPs and FD500 as cargo molecule. for the BBD with extend variable ranges. Triplicates were performed to assess model output reproducibility. Values indicated in red and with * are significant at $p < 0.05$.

	**BBD extended ranges**					
	**Replicate 1**		**Replicate 2**		**Replicate 3**	
**Term**	**Estimate**	**p-value**	**Estimate**	**p-value**	**Estimate**	**p-value**
Intercept	42.181	$5.891e^{-9}$ *	45.123	$4.620e^{-8}$ *	41.616	$3.67e^{-6}$ *
Size	4.961	$2.199e^{-4}$ *	3.882	0.006 *	6.954	0.009 *
Conc.	2.597	0.006 *	2.104	0.069	3.299	0.121
Fluence	-2.997	0.003 *	-2.467	0.041 *	$-3.953e^{-5}$	0.999
Size*Conc.	-5.189	0.001 *	-7.723	0.001 *	-4.996	0.102
Size*Fluence	-2.943	0.016 *	-5.435	0.007 *	-3.055	0.282
Conc.*Fluence	-5.990	$5.218e^{-4}$ *	-5.065	0.009 *	-3.302	0.249
Size²	-18.443	$8.188e^{-7}$ *	-17.338	$1.327e^{-5}$ *	-17.684	$4.819e^{-4}$ *
Conc.²	-5.758	$6.447e^{-4}$ *	-6.668	0.003 *	-5.500	0.078
Fluence²	-4.352	0.003 *	-3.543	0.039 *	-3.250	0.256
**Statistic**	**F-value**	**p-value**	**F-value**	**p-value**	**F-value**	**p-value**
Model	76.46	$1.692e^{-5}$ *	32.27	$2.107e^{-4}$ *	8.585	0.008 *
Lack-of-fit	1.331	0.410	0.671	0.625	5.239	0.104

Table S8. Summary statistics for the optimization of photoporation yield using PDNPs as photothermal NPs and FD500 as cargo molecule. for the BBD with extended variable ranges. Triplicates were performed to assess model output reproducibility. Values indicated in red and with * are significant at $p < 0.05$ for a one-sided unpaired Welch's t-test between the CCC design- and revised BBD-based model summary statistics.

	**BBD extended ranges**			
	**Replicate 1**	**Replicate 2**	**Replicate 3**	**Mean ± standard deviation**
**Statistic**	**Value**	**Value**	**Value**	**N.A.**
Multiple R^2	0.993	0.980	0.928	0.967 ± 0.034 *
Adjusted R^2	0.978	0.949	0.820	0.916 ± 0.084 *
PRESS	187	324	2204	905 ± 1127
Predicted R^2	0.914	0.848	0.011	0.591 ± 0.503
RMSE	1.09	1.64	3.17	1.967 ± 1.078 *
MAE	0.915	1.20	2.90	1.672 ± 1.073 *
**Test**	**p-value**	**p-value**	**p-value**	**N.A.**
Shapiro-Wilk	0.995	0.139	0.386	**N.A.**
Bruesch-Pagan	0.713	0.723	0.082	**N.A.**
Durbin-Watson	0.228	0.170	0.544	**N.A.**

Table S9. Summary of the stationary points (S.P.) of all replicates for the BBD with extended variable ranges in original units. yield at the S.P. coefficients of variation (C.V.) and all corresponding eigenvalues. providing more information on the nature of the S.P.

	**BBD extended ranges**			
	**Replicate 1**	**Replicate 2**	**Replicate 3**	**C.V.**
**Size (nm)**	523.056	529.288	535.124	1.141
**Concentration (*10^8 NPs/ml)**	4.162	3.681	3.578	8.188
**Fluence (J/cm²)**	0.608	0.648	0.885	20.979
**Delivery yield at S.P. (%) with 95% confidence intervals**	44.394 ± 2.411	46.633 ± 3.307	42.703 ± 6.124	4.422
**Eigenvalues**	-1.965<br>-7.351<br>-19.237	-2.109<br>-6.125<br>-19.315	-2.375<br>-5.664<br>-18.396	N.A.

Figure S5. Representative response surfaces where one factor was kept constant and the other factors varied. Regardless of the (A) PDNP concentration. (B) PDNP size or (C) laser fluence that was kept constant. the BBD with extended variable ranges did indicate the presence of an optimum.

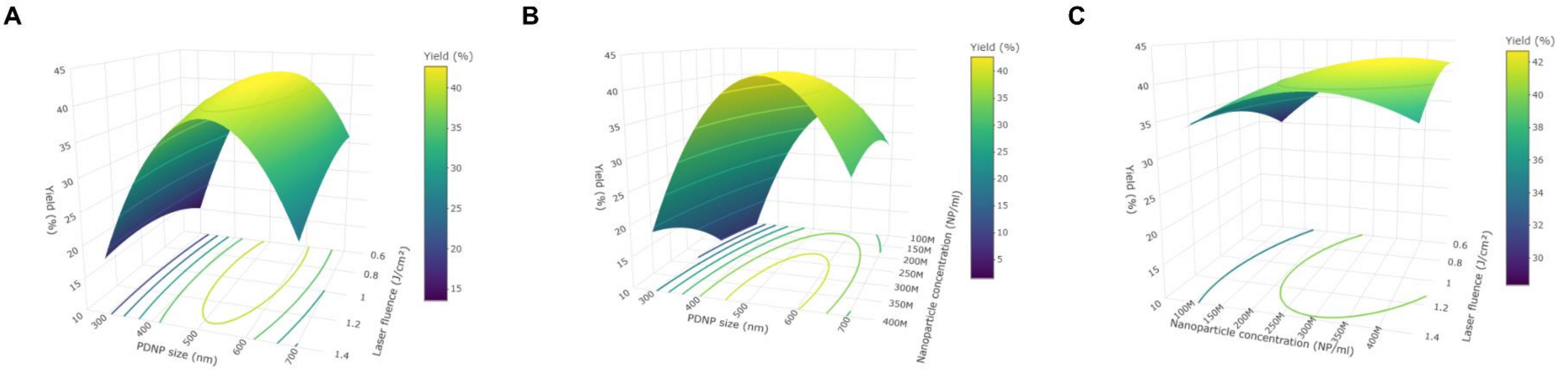

Table S10. Summary of the unpaired Welch's t-test between the averaged predicted values and averaged observed values for the CCC design-based models. Values indicated in red and with * are significant at $p < 0.05$.

	Variables			Observed values		Predicted values		t-test	
**Run**	**Size**	**Concentration**	**Fluence**	**Mean**	**SD**	**Mean**	**SD**	**t-value**	**p-value**
1	0.25	0.5	0.5	29.437	1.902	36.157	3.268	-3.078	0.049 *
2	-0.5	-0.5	0.5	24.316	7.639	30.948	3.113	-1.392	0.269
3	-0.5	0.5	-0.5	24.166	5.488	33.829	2.735	-2.730	0.074
4	0.25	-0.5	-0.5	39.794	4.459	37.915	3.079	0.600	0.584
5	0.25	0.5	-0.5	34.062	1.541	37.943	3.099	-1.942	0.150
6	0.25	-0.5	0.5	32.018	4.620	36.450	2.686	-1.436	0.241
7	-0.5	0.5	0.5	28.528	5.513	32.537	3.742	-1.042	0.364
8	-0.5	-0.5	-0.5	20.523	3.739	31.919	2.600	-4.334	0.016 *
9	-1	0	1	19.692	7.325	20.704	3.781	-0.213	0.845
10	-1	-1	0	13.683	4.520	17.692	3.134	-1.262	0.283
11	0	-1	-1	36.334	9.907	32.200	3.537	0.681	0.554
12	1	1	0	23.224	5.012	26.491	3.657	-0.912	0.418
13	0	1	-1	38.735	4.971	33.831	2.948	1.470	0.231
14	1	-1	0	27.956	5.852	30.519	3.189	-0.666	0.552
15	1	0	-1	29.069	2.528	31.436	3.801	-0.898	0.427
16	0	-1	1	39.573	7.950	29.919	2.739	1.988	0.160
17	-1	0	-1	16.669	4.824	22.309	1.001	-1.983	0.176
18	-1	1	0	19.866	5.767	23.699	4.592	-0.901	0.421
19	1	0	1	22.230	0.432	27.197	1.298	-6.287	0.015 *
20	0	1	1	36.720	2.177	30.268	4.369	2.290	0.108

Table S11. Summary of the unpaired Welch's t-test between the averaged predicted values and averaged observed values for the BBD-based models using a BBD with extended PDNP concentration and laser fluence ranges. Values indicated in red and with * are significant at $p < 0.05$.

	Variables			Observed values		Predicted values		t-test	
Run	Size	Concentration	Fluence	Mean	SD	Mean	SD	t-value	p-value
1	0.25	0	0.3	29.437	1.902	42.009	1.414	-9.188	0.001 *
2	-0.5	-0.5	0.3	24.316	7.639	31.974	2.425	-1.655	0.219
3	-0.5	0	-0.3	24.166	5.488	35.525	2.762	-3.203	0.050
4	0.25	-0.5	-0.3	39.794	4.459	40.875	2.152	-0.378	0.731
5	0.25	0	-0.3	34.062	1.541	43.674	1.918	-6.766	0.003 *
6	0.25	-0.5	0.3	32.018	4.620	40.646	1.653	-3.045	0.070
7	-0.5	0	0.3	28.528	5.513	35.576	2.628	-1.999	0.144
8	-0.5	-0.5	-0.3	20.523	3.739	30.488	2.434	-3.869	0.024
9	-1	0.25	0.6	19.168	7.338	20.810	4.256	-0.335	0.758
10	1	-0.25	0	26.262	4.842	30.870	1.835	-1.541	0.236
11	-1	0.25	-0.6	19.090	7.274	19.858	3.725	-0.163	0.881
12	-1	-0.75	0.6	13.305	5.476	12.058	3.079	0.344	0.753
13	0	-0.25	0.6	23.111	8.938	40.221	1.782	-3.252	0.075
14	0	-0.25	-0.6	38.841	2.164	40.971	2.455	-1.127	0.324
15	1	0.25	0.6	20.239	3.217	23.784	2.555	-1.494	0.213
16	1	0.25	-0.6	28.705	1.443	31.978	1.844	-2.421	0.076
17	-1	-0.75	-0.6	15.425	2.595	5.363	2.138	5.183	0.007 *
18	-1	-0.25	0	22.932	9.079	17.354	3.324	0.999	0.404
19	0	-0.75	0	34.199	10.266	37.612	1.948	-0.566	0.625
20	0	0.25	0	37.972	5.081	43.266	1.713	-1.710	0.206
21	1	-0.75	-0.6	34.704	3.256	29.422	3.459	1.926	0.127
22	1	-0.75	0.6	28.532	3.118	26.970	1.784	0.753	0.503

Table S12. Summary of key statistics in the comparison of the observed values of the confirmatory runs with the models based on both the CCC design and BBD (both original design and extended range design) for three factors and four center runs. The models' performance is considerably weaker when using the confirmatory run data. This evidenced by both the consistent decrease in R^2-values and increase in RMSE- and MAE-values when compared to the original model output.

	**CCC**			**BBD**			**BBD extended ranges**		
	**Replicate 1**	**Replicate 2**	**Replicate 3**	**Replicate 1**	**Replicate 2**	**Replicate 3**	**Replicate 1**	**Replicate 2**	**Replicate 3**
**R^2**	0.431	0.412	0.373	0.382	0.405	0.466	0.458	0.468	0.454
**RMSE**	8.626	7.174	7.056	8.326	9.609	8.101	8.156	9.721	8.351
**MAE**	7.423	5.970	5.516	6.504	7.534	6.396	6.552	7.995	6.696

Figure S6. Comparison of the yield its constituent parts (delivery efficiency & relative viability) obtained at different factor level combinations. Yield deconstructed in delivery efficiency and relative viability. revealing the fundamental reason for the existence of an optimum in the photoporation process. Error bars = standard deviations.

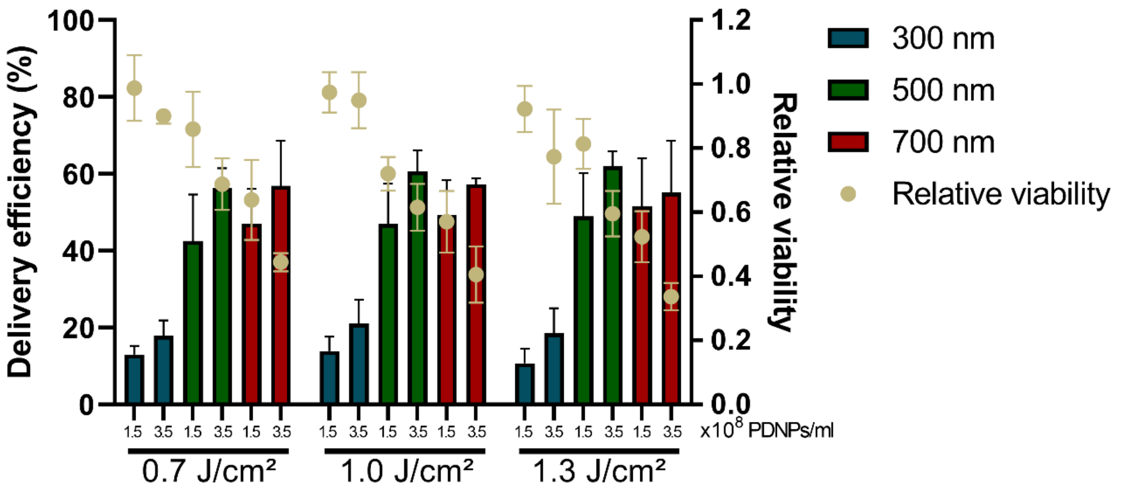

Table S13. Three-factor Circumscribed Central Composite (CCC) design and Box-Behnken Design (BBD), both with randomized run orders and four center-point runs, for the optimization of delivery yield of FITC-dextran 500 kDA (FD500) delivery in RAW264.7 murine macrophage-like cells. Factors used in this study were polydopamine nanoparticle (PDNP) size, PDNP concentration and laser fluence. Coded factor levels are presented.

	**CCC**			**BBD**		
**Run number**	**PDNP size**	**PDNP concentration**	**Laser fluence**	**PDNP size**	**PDNP concentration**	**Laser fluence**
**1**	0	0	0	-1	0	1
**2**	-1	1	1	0	0	0
**3**	1.789	0	0	-1	-1	0
**4**	-1	1	-1	0	-1	-1
**5**	-1	-1	1	1	1	0
**6**	0	0	1.789	0	1	-1
**7**	0	0	0	1	-1	0
**8**	0	0	-1.789	0	0	0
**9**	1	1	1	0	0	0
**10**	1	1	-1	1	0	-1
**11**	-1	-1	-1	0	-1	1
**12**	0	0	0	-1	0	-1
**13**	-1.789	0	0	-1	1	0
**14**	0	-1.789	0	1	0	1
**15**	0	1.789	0	0	1	1
**16**	1	-1	-1	0	0	0
**17**	0	0	0			
**18**	1	-1	1			

Table S14. Eight confirmatory runs within the design space of interest used to validate the RSM model outputs. Factors used in this study were polydopamine nanoparticle (PDNP) size, PDNP concentration and laser fluence. Coded factor levels are presented.

	**Confirmation runs**		
**Run number**	**PDNP size**	**PDNP concentration**	**Laser fluence**
**1**	0.25	0.5	0.5
**2**	-0.5	-0.5	0.5
**3**	-0.5	0.5	-0.5
**4**	0.25	-0.5	-0.5
**5**	0.25	0.5	-0.5
**6**	0.25	-0.5	0.5
**7**	-0.5	0.5	0.5
**8**	-0.5	-0.5	-0.5
